# Effects of remote ischemic postconditioning on the pro-inflammatory neutrophils of peripheral blood in acute cerebral infarction

**DOI:** 10.18632/aging.204751

**Published:** 2023-05-30

**Authors:** Zhen Liang, Lin Qiu, Xu Wang, Liangshu Feng, Yulei Hao, Feng Yang, Di Ma, Jiachun Feng

**Affiliations:** 1 Department of Neurology and Neuroscience Center, The First Hospital of Jilin University, Changchun, China; 2 Department of Neurology, China-Japan Union Hospital of Jilin University, Changchun, China

**Keywords:** acute cerebral infarction, neutrophils, pro-inflammatory neutrophils, remote ischemic postconditioning

## Abstract

Background: Neutrophils play crucial roles in the inflammatory response after acute cerebral infarction (ACI). Previous studies revealed neutrophils are non-homogeneous and can be divided into at least two subtypes, pro-inflammatory and anti-inflammatory, correlated with patients’ prognosis.

Objective: We aimed to explore the correlation between disease severity and peripheral blood neutrophils in patients with ACI and determine whether remote ischemic postconditioning (RIPostC) exerts neuroprotective effects by regulating neutrophils.

Methods: Patients (*n* = 38) with acute anterior circulation cerebral infarction were assigned to conventional treatment (*n* = 24; included aspirin, statins, neuro nutrition drugs, and circulation improvement drugs) or RIPostC (*n* = 14; 7-day ischemia adaptation [complete ischemia of both upper extremities for 5 minutes followed by remission for 5 minutes, 5 repeated cycles, twice a day, started from the morning of the second day of admission] based on conventional treatment) groups, based on their preference. General clinical data and peripheral blood samples were taken three times, in the morning before and 3 and 7 days after treatment. Fifteen adults with non-acute cerebral infarction matched for sex, age, and risk factors were recruited as controls; peripheral blood samples were only collected on the recruitment day. We used flow cytometry to detect the percentage of neutrophils and Real-Time PCR to detect the gene expression of interleukin (IL)-1β in the peripheral blood samples.

Results: The percentage of neutrophils, pro-inflammatory neutrophils (IL-1β high expression in flow cytometry), and IL-1β mRNA expression increased after ACI (*P* = 0.01, *P* = 0.001, *P* < 0.001). The National Institutes of Health Stroke Scale (NIHSS) score of patients with ACI within one day of onset was positively correlated with the percentage of pro-inflammatory neutrophils (*R* = 0.618, *P* = 0.043). Pro-inflammatory neutrophils in the RIPostC group decreased compared with those in the conventional treatment group, with the most significant difference observed on Day 7 (*P* = 0.01). However, the percentage of neutrophils was not statistically different. IL-1β mRNA expression decreased, with the most significant difference on Day 3 (*P* = 0.004). The NIHSS and Modified Rankin Scale scores for RIPostC decreased more significantly than for conventional treatment (*P* = 0.002, *P* = 0.019).

Conclusion: More severe cerebral infarction was associated with a higher percentage of pro-inflammatory neutrophils. The neuroprotective effect of RIPostC may partly be exerted through gene regulation to reduce pro-inflammatory neutrophils.

## INTRODUCTION

Acute cerebral infarction (ACI) is a multi-factorial ischemic cerebrovascular disease with extremely high mortality and morbidity. ACI, cardiovascular disease, and malignant tumors are the three major causes of human death, greatly increasing medical and societal burdens [1]. The current clinical treatment for cerebral infarction is limited to recanalization during the acute phase, including intravenous thrombolysis and mechanical thrombectomy [2, 3]. Although a certain curative effect has been achieved, studies revealed that cerebral ischemia/reperfusion (I/R) causes secondary injury, further expanding the scope of damage after cerebral infarction and resulting in more serious neurological deficits [2]. I/R injury after cerebral infarction involves multiple mechanisms, including calcium overload, amino acid toxicity, oxidative stress, and immune mechanisms [4]. Among them, the dual role of the immune response in the different phases of ACI has garnered increasing attention. In the acute phase of ACI, immune cells mainly initiate neuronal death by inducing glutamate toxicity and releasing cytotoxic chemokines and pro-inflammatory cytokines (potentially by microglia, but also likely involving neurons and glia cells). In the chronic phase of ACI, immune cells eventually exert phagocytic functions to facilitate the removal of dead cells and debris, promoting tissue repair [5, 6].

Neutrophils are the most abundant immune cells in peripheral blood, playing important roles in the inflammatory response after ACI. Some studies revealed that neutrophils rapidly increased and migrated to the central nervous system (CNS) after ACI [7–9]. Cai et al. [10] established a mouse model of middle cerebral artery occlusion (MCAO) and reported that the levels of peripheral blood neutrophil chemokines (including CXCL1, CXCL2, CXCL3 and CXCL5) rapidly increase after cerebral I/R, reaching a peak at 12 h. The level of neutrophils in the peripheral blood and spleen increased rapidly after cerebral ischemia and peaked at 24 h. Gelderblom et al. [11] observed in mouse MCAO models that neutrophils in peripheral blood migrate, adhere and infiltrate from the periphery to the CNS after I/R and reach a peak of infiltration at 3 days. Neutrophils infiltrating the CNS can produce proinflammatory mediators [such as interleukin (IL)-1β] and a series of destructive factors, including reactive oxygen species (ROS), neutrophil elastase, and matrix metalloproteinases 9 (MMP-9), which leads to destruction of the blood-brain barrier (BBB), further aggravating inflammatory injury after ischemia in ACI patients [12, 13]. Jickling et al. [14] found that neutrophil depletion after cerebral infarction can help reduce infarct size and improve the prognosis of ACI patients. Grønberg et al. [15] reported that inflammation contributes to removing necrotic neurons in the ischemic core and promotes astroglia proliferation, which exerts neuroprotective effects in animal stroke models. These differences suggest neutrophils play a dual role in the neuroinflammatory response after cerebral ischemia. Studies have shown that similar to macrophages, neutrophils can undergo phenotypic transformation after stimulation. Cai et al. [10] analogized the typing of macrophages and defined CD11b+ Ym1+ as N2-type (anti-inflammatory) neutrophils, and defined CD11b+ Ym1- as N1-type (pro-inflammatory) neutrophils in animal stroke models. Gordon et al. [16] observed in animal stroke models that N2-type neutrophils can play a neuroprotective role after cerebral ischemia by secreting and expressing Ym1, which may be related to extracellular matrix reorganization, but the exact mechanism requires further study. Compared with the N1-type, N2-type neutrophils are more capable of phagocytosing, clearing debris from damaged tissues, and expressing more anti-inflammatory factors including CD206, Arginase1, IL-10 and TGF-β [10]. This is beneficial in restoring tissue homeostasis and improving stroke outcomes. It can be concluded from the above that neutrophils are not homogeneous, and can be divided into at least two subtypes, pro-inflammatory and anti-inflammatory neutrophils. However, ideal markers to distinguish the two subtypes have not been identified. According to our previous tests described below, IL-1β is a classic pro-inflammatory mediator and one of the key modulators of the inflammatory response in cerebral I/R injury. IL-1β can promote the expression of adhesion molecules and cytokines, accelerate the migration of inflammatory cells to the lesion, and aggravate the injury after cerebral ischemia [17]. Therefore, we referred to cells with high expression of IL-1β as pro-inflammatory neutrophils.

Remote ischemic postconditioning (RIPostC), a non-invasive physical therapy method, can exert neuroprotective effects after a stroke [18]. The body can mobilize its endogenous protective mechanism to protect the vital organs when the upper or lower limbs receive repeated, short-term, non-lethal ischemic adaptation training. For cerebral infarction, studies have shown that RIPostC can protect the brain by protecting the BBB and improving cerebral edema [19]. Besides, an animal experiment confirmed that RIPostC could reduce neuronal death in a rat ischemia model, thereby reducing infarct size and improving prognosis [20]. For clinical patients, RIPostC can improve the prognosis of patients with ACI by increasing HSP27 [21]. RIPostC can also reduce the recurrence rate of cerebral infarction by improving cerebral blood perfusion arteries in patients with cerebral vascular stenosis (caused by atherosclerosis) [22]. The mechanisms underlying the neuroprotective effects of RIPostC mainly include immune regulation, neural pathways, humoral circulation, and cerebral blood flow regulation. In recent years, there has been increasing focus on the immune response after cerebral infarction, and RIPostC can improve the neurological deficit in patients by regulating the immune response. However, the exact molecular mechanism requires further exploration.

In this study to clarify the correlation between disease severity and peripheral blood neutrophils in patients with ACI, further explore whether RIPostC exerts neuroprotective effects by regulating neutrophils. We recruited 38 patients with ACI within 72 hours of onset (24 in the conventional treatment group and 14 in the RIPostC group) and 15 control individuals, measuring the percentage of peripheral blood pro-inflammatory neutrophils with high expression of IL-1β and detected the expression levels of the IL-1β gene at different time points. Meanwhile, the National Institutes of Health Stroke Scale (NIHSS) score on admission and 7 days after treatment and the Modified Rankin Scale score on admission and 3 months after discharge were evaluated to reflect disease severity and prognosis, thus observing the neuroprotective effect of RIPostC. Exploring the relationship between RIPostC and neutrophils is meaningful for providing new insights of the immune mechanism underlying the neuroprotective effect of RIPostC.

## MATERIALS AND METHODS

### Study cases of IL-1β

**Figure 1:**
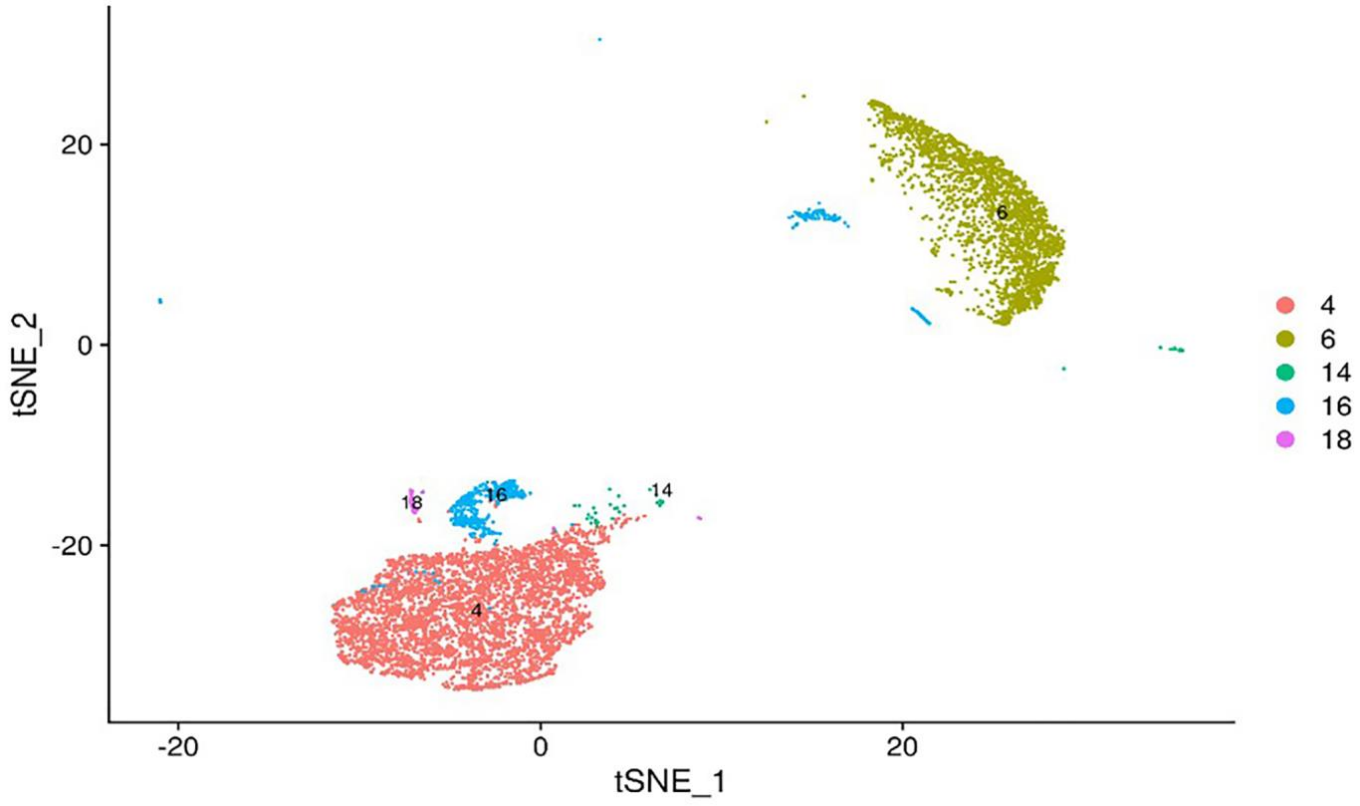
**tSNE map of mouse peripheral blood neutrophils.** Single-cell sequencing revealed that mouse peripheral blood neutrophils can be divided into 5 subsets: 4, 6, 14, 16, and 18. The potential gene markers of different neutrophil subsets are: IL-1β for subset 4, PbPb for subset 6, Wfdc17 for subset 14, Hbb-bt for subset 16 and Hdc for subset 18.

**Figure 2:**
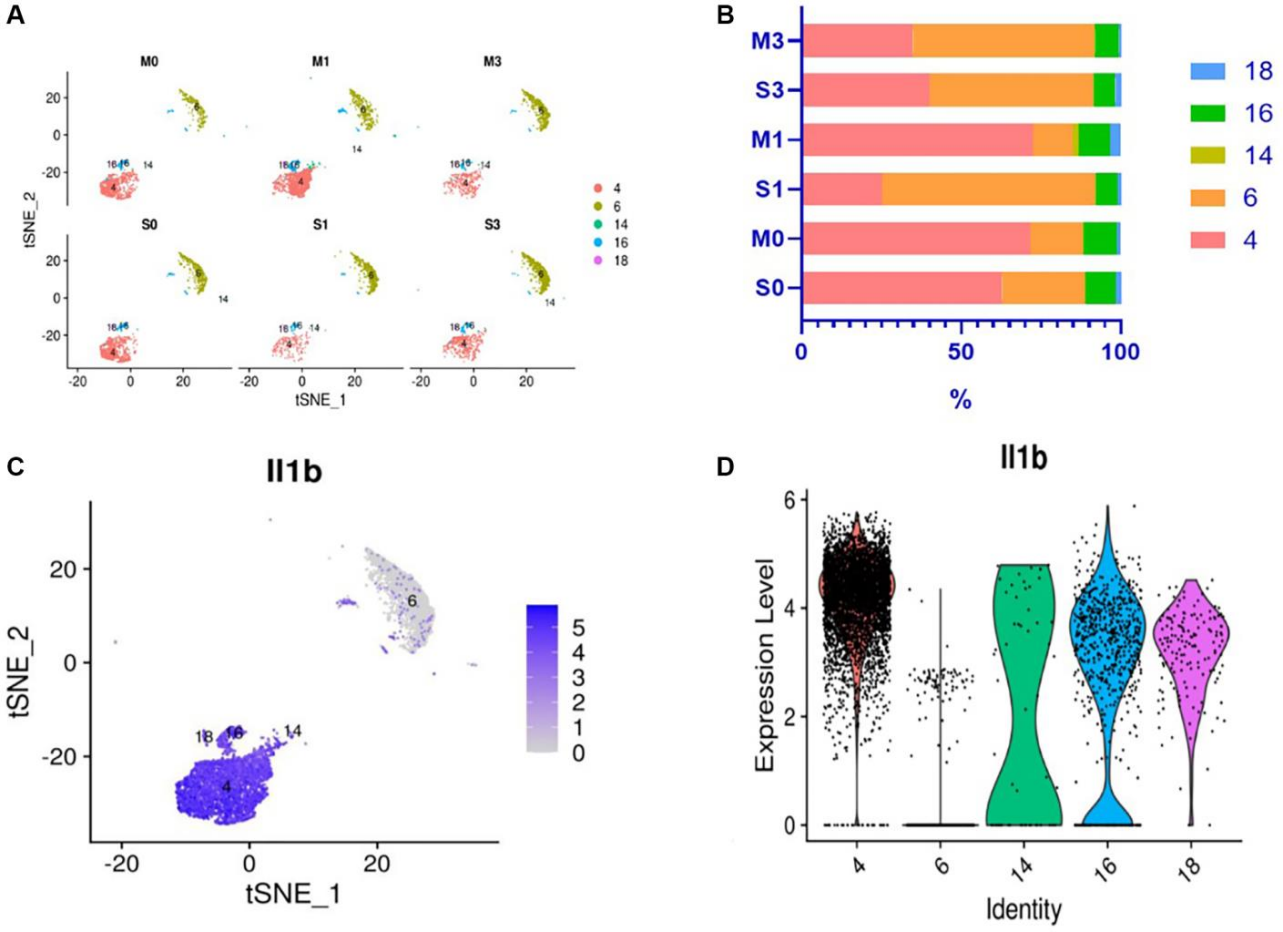
**Changes in neutrophil subsets and expression level of IL-1β gene in peripheral blood of mice after I/R.** Results of single-cell sequencing of peripheral blood neutrophils of mice: (**A**, **B**) Change trends and percentages of neutrophil subsets in peripheral blood of mice after ischemia/reperfusion (I/R). M0, M1, and M3 represent the mice in the experimental group whose blood was collected after cerebral ischemia and 24 and 72 h after cerebral I/R, respectively; S0, S1, and S3 represent sham-operated mice at the corresponding time points. It can be seen that neutrophil subset 4 in the peripheral blood of mice initially increased and then decreased after I/R. (**C**, **D**) Gene expression of IL-1β in different subsets of mouse peripheral blood neutrophils. (**C**) The percentage of neutrophils expressing IL-1β is the highest in neutrophil subset 4; (**D**) The IL-1β gene expression level of neutrophil subset 4 is considerably higher than that of other subsets.

In previous research, we performed single-cell sequencing on mouse peripheral blood leukocytes and observed that mouse peripheral blood leukocytes were divided into 21 subgroups, among which subgroups 4, 6, 14, 16, and 18 are neutrophils (Figure 1). Further cluster analysis of the five subgroups of neutrophils revealed that one subgroup of neutrophils had significantly higher levels of interleukin-1β (IL-1β) gene expression than others, which initially increases and then decreases after I/R (Figure 2). Therefore, the number of pro-inflammatory neutrophils can be determined by measuring the level of IL-1β expression in peripheral blood.

### Mouse

#### Reagents and equipment

We used the following equipment: R500 Universal Small Animal Anesthesia Machine (Shenzhen Reward Life Technology Company, China); Small Animal Surgical Microscope (Shanghai Yuyan Scientific Instrument Company, China); Immunomagnetic bead cell sorter (Miltenyi Biotec, Germany); Automatic cell counter (Thermo Fisher Scientific, USA); Ordinary thermal cycler (BIO-RAD, USA); Benchtop centrifuge (Eppendorf, Germany); Chloral hydrate (Dalian Meilun Biotechnology Company, Ltd., China); Red blood cell lysate (Beijing Soleibo Technology Company, China); Dead Cell Removal Kit (Miltenyi Biotec, Germany); Trypan Blue (Beijing Solebao Technology Company, China); Piperacillin and Sulbactam Sodium (Pfizer, USA).

The Animal Use and Care Committee approved all experimental procedures at the First Hospital of Jilin University.

#### Single-cell sequencing of mouse peripheral leukocyte

C57BL/6 mice (20–25 g, purchased from Jilin Qianhe Model Biotechnology Company, China) were divided into 6 groups (4–5 mice per group): 45 min cerebral ischemia group (I), 24 h cerebral ischemia-reperfusion group (I/R24h), 72 h cerebral ischemia-reperfusion group (I/R72h), and three sham-operated groups corresponding to each time point respectively. First, the mouse MCAO model was constructed. Second, the mouse peripheral leukocyte single cell suspension was prepared. Third, single-cell sequencing was performed with the support of technicians from Hangzhou Lianchuan Biotechnology Company, China. Subsequently, the single-cell sequencing data was analyzed, the differential expression of genes in different cell subsets was obtained, and the cell type was identified, relying on the Lianchuan biological cloud platform.

### Patient

#### Patient sample collection

##### Inclusion criteria

At the Department of Neurology of First Hospital of Jilin University from October 2017 to October 2019, patients with acute anterior circulation ischemic stroke (confirmed by diffusion-weighted imaging (DWI) within 72 h of onset who voluntarily consented to participate in the study were included, aged 18–80 years, regardless of sex, and National Institutes of Health Stroke Scale (NIHSS) score between 0–15.

##### Exclusion criteria

1. Co-infection before (<1 week) or after the onset;
2. Current use of steroids or immunosuppressants, or infected with human immunodeficiency virus, or combined with immune system diseases;
3. Definite cardiogenic embolism and diseases that may lead to embolism such as valve replacement, mural thrombus; congestive heart failure, severe arrhythmia;
4. Suspected intracranial arterial dissection, moyamoya disease, intracranial inflammatory vascular disease, vascular malformation, tumors and other neurological diseases; surgically treated intracranial lesions; received thrombolytic therapy;
5. Severe hypertension that is difficult to control (after drug treatment, systolic blood pressure >180 mmHg or diastolic blood pressure >110 mmHg);
6. Subclavian artery stenosis ≥50% or subclavian steal syndrome and upper extremity arterial occlusion;
7. Aspartate aminotransferase or alanine aminotransferase >3 times higher than the upper limit of normal (ULN); creatinine clearance rate <0.6 mL/s or serum creatinine >265 μmol/L (>3.0 mg/dL); severe blood system disease or severe coagulation dysfunction, platelet <100 × 10^9^/L;
8. Major cardiovascular surgery (such as cardiac, aortic or carotid surgery) within 30 days before and after enrollment;
9. Distant ischemia contraindicated, such as severe soft tissue injury, vascular injury, or upper extremity fracture.

##### Group therapy and sample collection

The group undergoing conventional treatment received plaque stabilization therapy, antiplatelet agents, treatments to improve circulation and nutrition, as well as symptomatic treatment. The RIPostC group, on the other hand, underwent ischemic adaptation training in addition to the basic drug therapy. The researcher began by assisting the patient in placing a cuff on their bare arm, positioning it about 1–2 cm above the elbow joint and wrapping it snugly against the skin. The cuff was fastened tightly enough that only a finger could be inserted between it and the arm. Next, the ischemic adaptation training was conducted on the patient’s upper limbs using the ischemia adaptor according to the predetermined procedure. Each cycle included 5 minutes of ischemia, during which blood flow to both upper limbs was interrupted, followed by 5 minutes of reperfusion, during which blood flow was restored. Each training session consisted of 5 cycles and was performed once in the morning and once in the evening for a duration of 1 week. The appropriate mode of treatment was selected based on the patient’s systolic blood pressure, with the aim of temporarily interrupting blood flow to the limbs when the cuff pressure exceeded the patient’s systolic blood pressure by 30 mmHg. In the RIPostC group, 5 mL of peripheral blood was collected from patients who fasted for 8 h before RIPostC and 3 and 7 days after RIPostC (recorded as 0d, 3d, and 7d), respectively. Blood from the routine treatment group was also collected at the above time points. Blood from the control group was only collected blood on the day of recruitment. Heparin anticoagulation tubes were used for blood collection, temporarily stored in a refrigerator at 4°C and sent for analysis within 2 h.

#### Clinical data collection

The clinical data of ischemic stroke patients and healthy individuals were collected. The Ethics Committee of the hospital approved the study (Approval Number: AF-IRB-029-06). The investigations were conducted in conformance with the Declaration of Helsinki. The CONSORT-style flowchart of the study was in Supplementary Figure 1.

#### Reagents and equipment

Ischemic Preconditioning Therapy Apparatus (Model: IPC-906; Beijing Renqiao Cardiovascular and Cerebrovascular Disease Prevention Research Nantong Company, China). BD FACS Calibur flow cytometer (BD Company, USA); Low-temperature high-speed centrifuge (Heraeus, Germany); Anti-CD11b-BB700 (Clone Number: M1/70), Anti-CD66-FITC (Clone Number: B1.1/CD66) and Anti-IL-1β-Alexa Fluor 647 (Clone Number: JK1B-1), (BD Company, USA); Multi-function microplate reader: (BioTek Company, USA); PCR instrument: (Bio-Rad Company, USA); QuantStudio 3 Real-Time PCR System: (Thermo Fisher Scientific Company, China); Various peripheral animal blood neutrophil isolation liquid kits: (Number: Solarbio.P9040. Beijing Soleibao Technology Company, China); MonScript™ RTIII All-in-One Mix with dsDNase Kit: (Wuhan Mona Biotechnology Company, China); MonAmp™ ChemoHS qPCR Mix Kit: (Wuhan Mona Biotechnology Company, China); Primers: (Shanghai Sangon Bioengineering Company, China).

#### RT-PCR for IL-1β mRNA measurement

The 3 mL of venous blood collected from each participant was placed in a green heparin sodium blood collection tube, temporarily stored in a refrigerator at 4°C, and sent to the laboratory for sample processing within 2 h. Neutrophils were extracted using various animal peripheral blood neutrophil isolation liquid kits according to the manufacturer’s instructions. For the cell layer, cells in the lower layer (neutrophils) were pipette into a 1.5 mL RNase EP tube and centrifuged at 12000 rpm for 5 min. The supernatant was discarded, and the cells were obtained. According to the manufacturer’s instructions, IL-1β mRNA levels in each group were determined using Real-Time PCR. Total RNA was extracted from the neutrophils obtained in the above step using Trizol reagent. RNA was reverse transcribed to cDNA using the MonScript™ RTIII All-in-One Mix with dsDNase Kit. Real-time PCR was performed with the QuantStudio 3 Real-Time PCR machine using the MonAmp™ ChemoHS qPCR Mix Kit. The relative quantitative analysis data of the studied genes were calculated according to −ΔCT (ΔCT = target gene CT value-GAPDH CT value). The primer sequences for the study gene were as follows:

GAPDH: forward, 5′-GGAGCGAGATCCCTCCAAAAT-3′
GAPDH: reverse, 5′-GGCTGTTGTCATACTTCTCATGG-3′
IL-1β: forward, 5′-TTCGACACATGGGATAACGAGG-3′
IL-1β: reverse, 5′-TTTTTGCTGTGAGTCCCGGAG-3′.

#### Flow cytometry for neutrophils and pro-inflammatory neutrophil measurement

**Figure 3:**
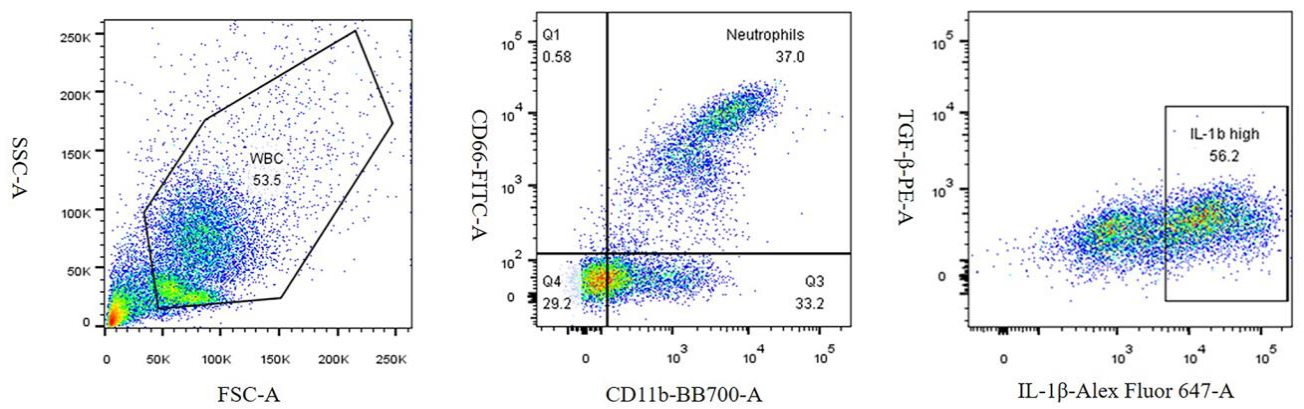
Density plots of neutrophils and pro-inflammatory neutrophils.

To determine the percentage of neutrophils in patients with ischemic stroke at different stages, we collected 2 mL of human peripheral blood in a heparin anticoagulation tube. According to the manufacturer’s instructions, 400 μL of blood, 100 μL of RPMI-1640 medium and 2 μL of stimulator were added to each flow tube. After mixing, it was incubated in a constant temperature incubator at 37°C for 5 h. After incubation, 3 times the erythrocyte lysis solution sample volume was added to the flow tube, pipetted evenly, lysed on ice for 15 min, and shaken every 5 min. The lysate was centrifuged at 1700 rpm for 5 min, and the supernatant was discarded. The pellet was re-lysed twice according to the ratio of erythrocyte lysate to samples 2:1 and 1:1. The entire procedure was conducted on ice. The steps were repeated, and finally, the supernatant was discarded. Subsequently, the cells were blocked in FACS buffer (1% fetal bovine serum in PBS) for 15 min and treated with anti-CD11b-BB700,anti-CD66-FITC, and anti-IL-1β-Alexa Fluor 647 antibodies for 30 min in the dark. The cells were then resuspended in 400 μL of flow cytometry staining buffer and analyzed using the BD FACS Calibur flow cytometer. Neutrophils were gated according to forward-angle scatter (FSC) and side-scattered light (SSC), 10,000 cells were collected per tube, and flow cytometry results were analyzed using Flowjo V10 software. CD11b+CD66+ cells were regarded as neutrophils [23]. IL-1β high expression cells in neutrophils were regarded as pro-inflammatory neutrophils (Figure 3).

#### Experimental group

Thirty-eight patients with acute anterior circulation cerebral infarction who fulfilled the inclusion criteria were assigned to the conventional treatment and the RIPostC groups according to their preferences. In addition, 15 non-acute cerebral infarction adults (healthy volunteers) who were recruited during the same period and matched for sex, age, and related risk factors were used as the control group. Finally, 24 patients were recruited in the conventional treatment group, 14 patients in the RIPostC group, and 15 volunteers in the control group. To analyze the correlation between the severity of illness and neutrophil typing at admission, all the patients with cerebral infarction were regrouped according to the time of onset and the time of first blood collection after admission, and they were divided into 1 d and 2–3 d groups. In order to explore whether the timing of the application of remote limb ischemia postconditioning impacts the treatment effect, patients in the RIPostC group were further divided into 1 d and 2–3 d groups according to the time of application of remote limb ischemia postconditioning.

### Statistical analysis

The data were analyzed using Statistical Product and Service Solutions (SPSS) version 22 (IBM, USA). Independent samples *t*-test and Pearson’s correlation were used for normally distributed data. The Mann–Whitney *U* test and Spearman’s correlation were used for data with non-normal distribution. *P* < 0.05 was considered statistically significant.

### Availability of data and materials

The datasets collected and/or analyzed during this study are available from the corresponding author on reasonable request.

## RESULTS

### Clinical baseline comparison before treatment

A total of 38 patients were included in the study, RIPostC (*n* = 14, mean age: 60.07 ± 13.49 years), conventional treatment (*n* = 24, mean age: 67.33 ± 9.37 years), and control (*n* = 15, mean age: 60.58 ± 14.96 years). In the RIPostC, control and the conventional treatment groups, the male to female ratios were 6:1, 4:1 and 1.2:1, respectively; hypertension and diabetes accounted for 42.9% vs. 40% vs. 54.1% and 42.9% vs. 53.3% vs. 41.7%, respectively; smokers accounted for 71.4% vs. 26.7% vs. 37.5%, respectively; and drinkers accounted for 57.1% vs. 46.7% vs. 37.5%, respectively. The above data were not statistically different between the control and conventional treatment groups (Table 1).

**Table 1:** Baseline data of patients in conventional treatment group and control group.

	**Control group (*n* = 15)**	**Conventional treatment group (*n* = 24)**	***P*-value**
Age (years)	60.58 ± 14.96	67.33 ± 9.37	0.106
Sex (male/female)	12/3	13/11	0.102
Hypertension (yes/no)	6/9	13/11	0.389
Diabetes (yes/no)	8/7	10/14	0.477
Smoking (yes/no)	4/11	9/15	0.488
Drinking (yes/no)	7/8	9/15	0.571

The average ages of the RIPostC and conventional treatment groups were 60.07 ± 13.49 vs. 67.33 ± 9.37 years, respectively. In the RIPostC and conventional treatment groups, the male and female ratios were 6:1 vs. 1.2:1, respectively. Hypertension and diabetes accounted for 42.9% vs. 54.2% and 42.9% vs. 41.7%, respectively. Smokers and drinkers accounted for 71.4% vs. 37.5% and 57.1% vs. 37.5%, respectively. In addition, there were no statistical differences in baseline characteristics regarding sex, age, hypertension, and diabetes, smoking, and drinking histories, WBC#, NE#, NE%, LY#, LY%, PLT, Cr, total cholesterol (TC), triglyceride (TG), low-density lipoprotein cholesterol (LDL-C), glucose (Glu), glycosylated hemoglobin (HbAlc), alanine aminotransferase (ALT), aspartate amino transferase (AST, prothrombin time (PT, activated partial thromboplastin time (APTT), international normalized ratio (INR), fibrinogen (FIB), folic Acid, high-sensitivity C-reactive protein (hs-CRP), homocysteine (HCY), vitamin B12 (VitB12), NIHSS and mRS score on admission (Table 2).

**Table 2:** Baseline data of patients in the RIPostC group and the conventional treatment group.

	**Conventional treatment group (*n* = 24)**	**RIPostC group (*n* = 14)**	***P*-value**
Age (years)	67.33 ± 9.37	60.07 ± 13.49	0.058
Sex (male/female)	13/11	12/2	0.077
Hypertension (yes/no)	13/11	6/8	0.501
Diabetes (yes/no)	10/14	6/8	0.943
Smoking (yes/no)	9/15	10/4	0.111
Drinking (yes/no)	9/15	8/6	0.240
WBC (10^9^/L)	7.74 ± 2.04	6.70 ± 1.68	0.126
NE (10^9^/L)	5.22 ± 2.10	4.48 ± 1.46	0.258
NE (%)	65.93 ± 10.91	64.03 ± 11.69	0.624
LY (10^9^/L)	1.81 ± 0.64	1.78 ± 0.0.64	0.881
LY (%)	24.81 ± 9.75	27.40 ± 10.40	0.457
TG (mmol/L)	1.66 ± 0.89	1.61 ± 0.67	0.864
TC (mmol/L)	4.79 ± 1.15	4.73 ± 0.93	0.877
LDL-C (mmol/L)	3.00 ± 0.92	3.13 ± 0.70	0.656
Glu (mmol/L)	6.05 ± 1.19	6.87 ± 2.59	0.193
HbA1c (%)	6.50 (6.05–7.15)	5.70 (5.33–8.25)	0.159
AST (U/L)	21.90 (17.30–27.85)	19.90 (16.83–35.46)	0.582
ALT (U/L)	18.20 (12.50–22.10)	13.50 (11.18–20.63)	0.838
Cr (μmol/L)	69.40 ± 19.65	69.22 ± 11.54	0.976
PLT (10^9^/L)	208.50 ± 58.92	212.14 ± 49.14	0.847
PT (s)	11.44 ± 0.70	11.47 ± 0.91	0.911
APTT (s)	26.2 (24.3–27.15)	24.4 (23.65–27.55)	0.560
INR	0.94 ± 0.07	0.94 ± 0.09	0.968
FIB (g/L)	3.46 ± 1.22	3.19 ± 0.65	0.459
HCY (μmol/L)	11.92 (9.51–20.42)	14.95 (11.49–15.96)	0.082
Folic Acid (nmol/L)	5.93 ± 3.48	5.39 ± 2.80	0.627
Vit B12 (pmol/L)	338.08 ± 213.54	235.85 ± 77.21	0.106
NIHSS on admission	4.00 (2.00–7.75)	7.50 (4.00–9.00)	0.059
mRS on admission	4.00 (2.00–5.00)	5.00 (4.00–5.00)	0.071

### Comparison of neutrophil subsets between conventional treatment and control groups

**Figure 4:**
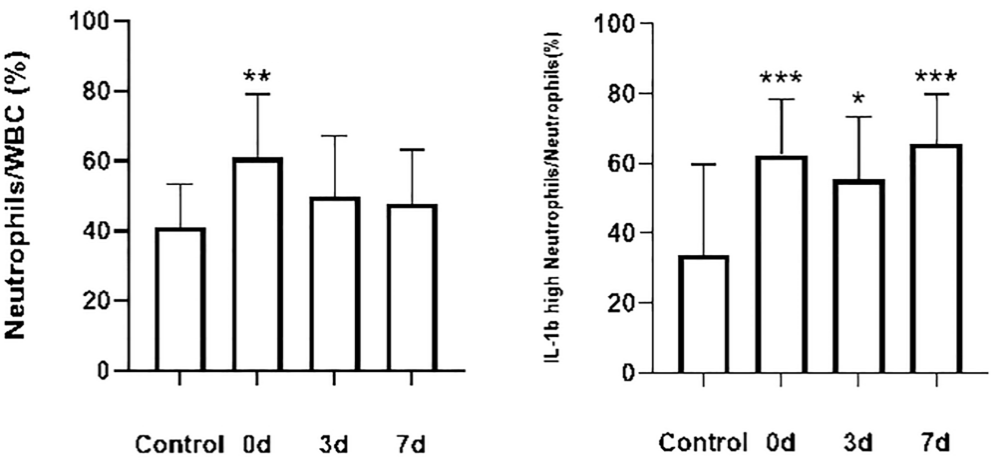
**Changes in the percentage of neutrophils and the percentage of pro-inflammatory neutrophils in the conventional treatment group compared with the control group.** Control group (*n* = 15), conventional treatment group (*n* = 24). Data from both groups were normally distributed. Differences between control and conventional treatment groups were compared using an independent samples *t*-test. Paired samples *t*-tests were used to compare the proportion of cells in each group at each time point in the conventional treatment group. ^*^P < 0.05 ^**^P < 0.01 ^***^P < 0.001.

To clarify the changes in peripheral blood neutrophil subsets in patients with ACI, patients in the conventional treatment group were analyzed, and the neutrophil subsets detected in the peripheral blood of the control group were compared. The results were as follows: Compared with the control group, the first blood collection after admission in the conventional treatment group showed significantly higher neutrophils and pro-inflammatory neutrophils (*P* = 0.01, *P* = 0.001). Subsequently, the neutrophils and pro-inflammatory neutrophils in the conventional treatment group exhibited a downward trend but did not reach statistical significance (Figure 4).

### Comparison of the expression level of IL-1β mRNA between the conventional treatment group and control group

**Figure 5:**
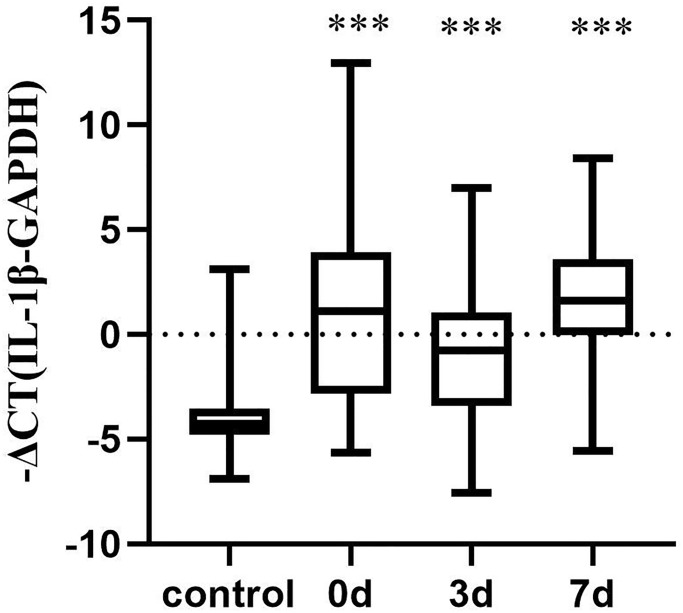
**Changes in IL-1β mRNA levels between the conventional and control treatment groups.** Control group (*n* = 15), conventional treatment group (*n* = 24). The data were not normally distributed. Differences between control and conventional treatment groups were compared using Mann Whitney *U*-test. Wilcoxon test was used to compare the proportion of cells in each group at each time point in the conventional treatment group. ^***^P < 0.001.

To clarify changes in IL-1β mRNA expression in the peripheral blood of patients with acute cerebral infarction, we used patients in the conventional treatment group as the research object and compared them with the control group. The relative quantification of IL-1β mRNA was represented by −ΔCT. The results are as follows: Compared with the control group, the IL-1β mRNA in the conventional treatment group was significantly increased (*P* < 0.001), and then showed a downward trend, but did not achieve statistical significance (Figure 5).

### The difference in treatment effect between RIPostC and conventional treatment groups

**Figure 6:**
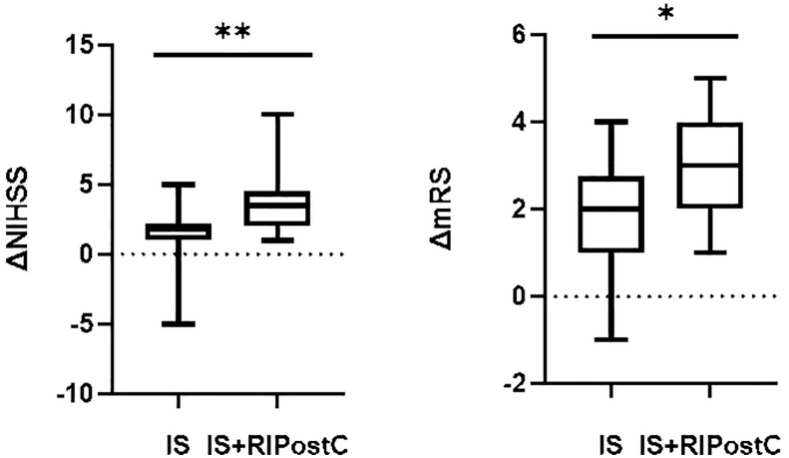
**Comparison of scores between RIPostC and conventional treatment groups.** Conventional treatment group (IS, *n* = 24), RIPostC group (IS+RIPostC, *n* = 14). The data were not normally distributed. The difference between the two groups was compared using Mann Whitney *U*-test. ^*^P < 0.05 ^**^P < 0.01.

The NIHSS score change (ΔNIHSS) after 7 days of treatment was used to measure the improvement in neurological deficits in the acute stage, and the change in the modified Rankin Scale (ΔmRS) score 3 months after discharge was used to determine the prognosis. The results showed that there was no significant difference in NIHSS and mRS scores between the two groups before treatment (*P* > 0.05). After treatment, the NIHSS and mRS scores of the two groups improved significantly compared to the pre-treatment values; however, improvement in the RIPostC group was more pronounced. (ΔNIHSS: *P* = 0.002, ΔmRS: *P* = 0.019) (Table 3, Figure 6).

**Table 3:** Comparison of patient scores before and after treatment in RIPostC and conventional treatment groups.

**Group**	**Score**	**Before treatment**	**After treatment**	***P*-value**
Conventional treatment group	NIHSS	4 (2–7.75)	1 (1– 4.75)	0.001
	mRS	4 (2–5)	1 (1–3)	<0.001
RIPostC group	NIHSS	7.5 (4–9)	2 (0.75–5)	0.001
	mRS	5 (4–5)	1.5 (1–3)	0.001

The two data groups are represented by M (P25-P75) for not normally distributed. Conventional treatment group (*n* = 24), RIPostC group (*n* = 14). The difference between the two groups before and after treatment was compared using the Wilcoxon test.

### Correlation between peripheral blood neutrophil subsets and disease severity in patients with acute cerebral infarction

**Figure 7:**
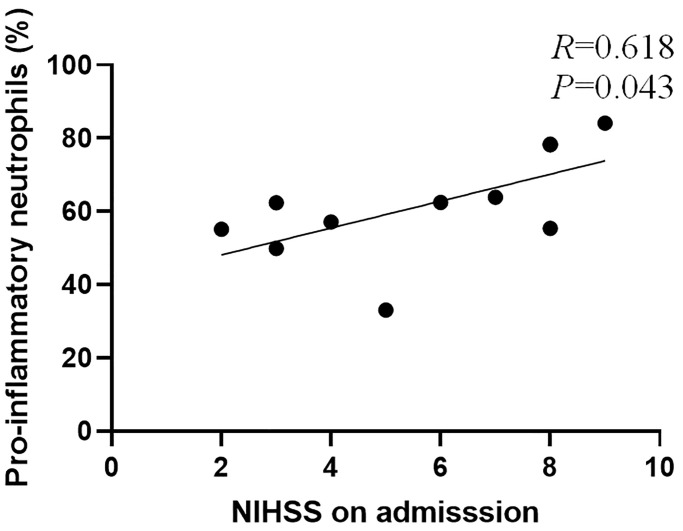
Scatter plot of NIHSS score and percentage of pro-inflammatory neutrophils.

Among the 38 patients with acute cerebral infarction evaluated in this study, 11 patients who completed the first blood collection within 1 day of onset were selected. The correlation between the NIHSS score and the percentage of peripheral blood neutrophils and pro-inflammatory neutrophils was analyzed. The NIHSS score at admission was positively correlated with the percentage of pro-inflammatory neutrophils, but there was no significant correlation with the percentage of neutrophils. The percentage of pro-inflammatory neutrophils was tested for normality, and displayed normal distribution (*P* > 0.05); Pearson correlation analysis was used. The results revealed that the correlation coefficient of pro-inflammatory neutrophils was R = 0.618, *P* = 0.043, that is, the percentage of pro-inflammatory neutrophils had a moderately positive correlation with disease severity. A scatterplot of NIHSS scores and pro-inflammatory neutrophil levels is shown below (Figure 7).

### Comparison of neutrophil subsets between RIPostC and conventional treatment groups

**Figure 8:**
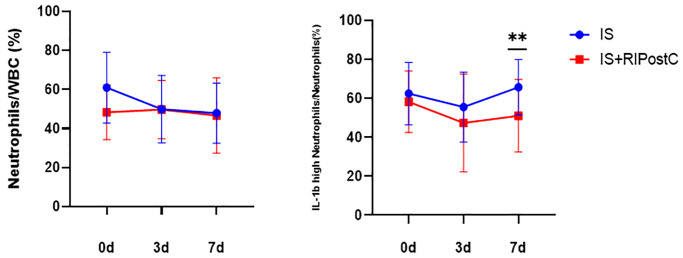
**Changes in the percentage of neutrophils and pro-inflammatory neutrophils in the RIPostC group compared with those in the conventional treatment group.** Conventional treatment group (IS, *n* = 24), RIPostC group (IS+RIPostC, *n* = 14). Data from both groups were normally distributed. ANOVA was used to compare the differences in the percentage of cells between the two groups at each time point. ^**^P < 0.01.

To explore the effect of RIPostC treatment on peripheral blood neutrophil subsets in patients with acute cerebral infarction compared with conventional treatment, this study evaluated 24 patients in the conventional treatment and 14 in the RIPostC groups (recorded as IS and IS+RIPostC group). All fulfilled the inclusion and exclusion criteria. The percentage of peripheral blood neutrophils and pro-inflammatory neutrophils in the two groups were measured before treatment and after 3 and 7 days of treatment (denoted as 0d, 3d, and 7d). The results are as follows: Compared with the patients in the conventional treatment group, the percentage of peripheral blood neutrophils in the RIPostC group exhibited a downward trend, but there was no statistical difference (*P* > 0.05). Pro-inflammatory neutrophils also showed a downward trend, and the difference was significant at 7 d after RIPostC treatment (*P* = 0.01). (Figure 8).

### Comparison of the expression level of IL-1β mRNA between RIPostC and conventional treatment groups

**Figure 9:**
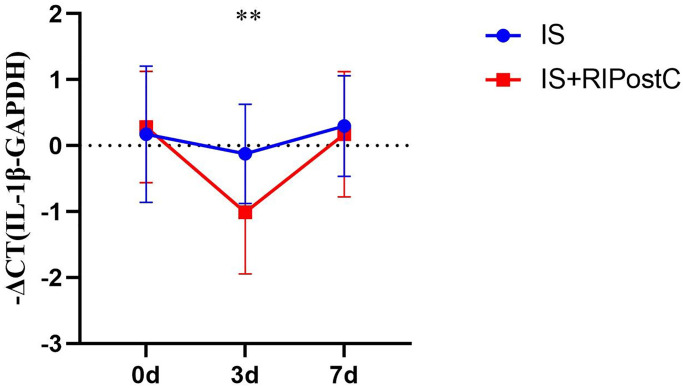
**Change in IL-1β mRNA levels between RIPostC and conventional treatment groups.** Conventional treatment group (IS, *n* = 24), RIPostC group (IS+RIPostC, *n* = 14). The data were not normally distributed. After normalization, ANOVA was performed to compare the differences in the percentage of cells between the two groups at each time point. ^**^P < 0.01.

To compare the changes of IL-1β mRNA expression level between RIPostC and conventional treatment groups, the relative quantification of mRNA was expressed by −ΔCT. −ΔCT did not conform to the normal distribution, and multivariate analysis of variance (ANOVA) was performed after normalization. The results were as follows: the IL-1β mRNA level in the RIPostC group was lower than that in the conventional treatment group and was most significantly decreased at 3 days post-treatment (*P* = 0.004, Figure 9).

## DISCUSSION

In this study, compared with the control group, the percentage of neutrophils in patients with acute cerebral infarction in the first blood collection after admission (the second day of admission) was significantly higher, then gradually decreased. This result is consistent with previous findings in animal models of ischemia. On the one hand, after the occurrence of cerebral ischemia, the hypothalamus-pituitary-adrenal gland (HPA) axis is activated, the secretion of adrenaline increases, and the number of neutrophils in the peripheral blood rapidly increases in a short period [24]. In a mouse ischemia model, Cai et al. [10] observed that the number of neutrophils in peripheral blood and spleen increased rapidly after cerebral ischemia, and the number of neutrophils in peripheral blood peaked at 24 h after ischemia. Another study found that neutrophils can reach the blood from the bone marrow through CXCR2 under the stimulation of chemokines released by injured tissues [25]. The results suggested that we can rapidly increase the number of circulating neutrophils quickly through HPA axis activation and bone marrow mobilization after cerebral infarction. On the other hand, injured tissue can recruit neutrophils from the periphery to the CNS by releasing chemokines such as CXCL1 and CXCL2/3 [26]. At the same time, injured tissue can also upregulate neutrophil surface adhesion receptors by releasing some inflammatory mediators, promoting adhesion to endothelial cells and migrating around the lesion [27]. In the mouse MCAO model, Gelderblom et al. found that the infiltration of peripheral blood neutrophils reached a peak from central infiltration in three days after I/R [11]. However, the role of neutrophils after cerebral infarction remains controversial. Studies suggest that the rapidly increasing neutrophils after cerebral ischemia can aggravate cerebral injury after ischemia by blocking capillaries in the brain [28], forming microthrombi [14], and producing a large number of pro-inflammatory mediators [13]. Some studies imply that the inflammatory process involved in neutrophils helps remove necrotic neurons and tissue debris and promotes gliosis, which has a protective effect [15]. The differences indicate that neutrophils do not play one role. This is not only related to the number of neutrophils; different phenotypes may play different roles. Recent studies have found that neutrophils can be activated into N1 and N2 types, similar to macrophages. The mainstream view is that the N1 type is pro-inflammatory, aggravating ischemic injury after cerebral infarction, whereas the N2 type is anti-inflammatory, which can remove debris and promote tissue repair, thereby improving ischemic injury [29]. However, there is still a lack of ideal markers to distinguish these, which limits further research. As previously mentioned, our research group found that IL-1β is suitable as a marker for pro-inflammatory neutrophils by single-cell sequencing of mouse peripheral blood neutrophils in animal experiments. Therefore, in this study, we found that the mRNA expression of IL-1β in the peripheral blood of patients in the conventional treatment group first increased and then decreased using qPCR compared with the control group, and both were higher than those in the control group. Furthermore, we designed a flow cytometry experiment that CD11b+CD66+IL-1βhigh was used to label pro-inflammatory neutrophils. The results showed that compared with the control group, the percentage of pro-inflammatory neutrophils was significantly increased in the patients in the conventional treatment group at three time points after admission. Similarly, there was a trend of initial decrease and subsequent increase when the three time points were compared, but the decrease was not statistically significant. Therefore, we can conclude that in the acute phase of cerebral infarction, the percentage of proinflammatory neutrophils in peripheral blood increases. This result is consistent with the findings of Kostulas et al. [30], who found that the IL-1β mRNA expression increased in most patients 1 to 3 days after onset of ACI and returned to the level of the healthy individuals after 20 to 31 days. Other studies have found that neutrophils can produce pro-inflammatory factors (such as IL-1β) and release a variety of proteases (including ROS and MMP-9) in the acute phase after ischemia, leading to the destruction of the BBB and further aggravating post-ischemic inflammatory damage [13, 15]. To explore the role of pro-inflammatory neutrophils, we compared the percentage of pro-inflammatory neutrophils with disease severity and prognosis. For 11 patients with acute cerebral infarction who could complete the first blood collection within 1 day of onset, the correlation between their NIHSS scores and the percentage of peripheral blood and pro-inflammatory neutrophils was analyzed. The results showed that the percentage of neutrophils in peripheral blood was moderately positively correlated with the severity of the disease; more severe disease showed higher levels of proinflammatory neutrophils. This indicates that pro-inflammatory neutrophils may aggravate ischemic cerebral injury, and the percentage of pro-inflammatory neutrophils in peripheral blood of patients with acute cerebral infarction of 1 day after onset can reflect the severity of the disease to a certain extent.

A total of 14 patients in the RIPostC group and 24 in the conventional treatment group were included in this study. According to the changes of NIHSS and mRS scores, the speed of neurological recovery and prognosis of the patients could be evaluated. It was found that the patients treated with RIPostC recovered faster and had better prognosis than those in the conventional treatment group, suggesting that RIPostC intervention has a therapeutic effect on patients with acute cerebral infarction, which is consistent with previous studies [31, 32]. The neuroprotective mechanisms of RIPostC treatment mainly include immune regulation, neural pathways, humoral circulation and cerebral blood flow regulation [33], in which the immune mechanism is closely related to neutrophils [34]. Among the 24 patients in the conventional treatment group and 14 patients in the RIPostC group included in this study, we measured the percentage of neutrophils in the peripheral blood and the percentage of proinflammatory neutrophils in the two groups before treatment, and 3 and 7 days after treatment. The results showed that compared with the conventional treatment group, the pro-inflammatory neutrophils in peripheral blood of the RIPostC group showed a downward trend, and the difference was significant at 7 days after RIPostC treatment. There was no statistical difference between the two groups. We also found that the mRNA expression of IL-1β in the peripheral blood of patients in the RIPostC group was lower than that in the conventional treatment group using Real-Time PCR. Combined with the above research results that pro-inflammatory neutrophils have a damaging effect after cerebral infarction, it can be concluded that the neuroprotective effect of RIPostC may be through reducing the percentage of pro-inflammatory neutrophils in patients with cerebral infarction in the acute phase. Studies have shown that RIPostC can inhibit peripheral and central neutrophils activation in rats [35]. Shimizu et al. [36] found that RIPostC could inhibit the activation of neutrophils, the adhesion to the endothelium and the expression of related inflammatory genes in humans. Likewise, Konstantinov et al. [37] found that RIPostC can reduce the expression of CD11b in humans. These studies can support our conclusions to a certain extent, but we still need to expand the sample size further. It should be noted that there were few studies on neutrophil typing after cerebral infarction, and there is a lack of adequate literature support. In addition, this study is an experiment with a small cohort. Moreover, only the percentage of pro-inflammatory neutrophils in peripheral blood is analyzed. For more comprehensive determination, it is necessary to conduct studies with larger sample sizes to confirm the above results. In this study, the neuroprotective effect of RIPostC in patients with acute cerebral infarction was related to the inflammatory response by observing the effect of RIPostC on the percentage of neutrophils in white cells and the percentage of neutrophils that are pro-inflammatory. Further, we analyzed the correlation between the percentage of pro-inflammatory neutrophils and the severity of cerebral infarction, which provides a basis for exploring the immune mechanism underlying the protective effect of RIPostC on the brain. This information could aid in the conducting targeted treatment for ACI with the potential to enhance patient outcomes greatly.

## Supplementary Materials

Supplementary Figure 1
