# Supplementary figures and images for: Effects of remote ischemic postconditioning on the pro-inflammatory neutrophils of peripheral blood in acute cerebral infarction
Source: Aging (Albany NY). 2023 May 30;15(10):4481–97. doi: 10.18632/aging.204751 (PMC10258025; doi:10.18632/aging.204751)

## SUPPLEMENTARY FIGURE

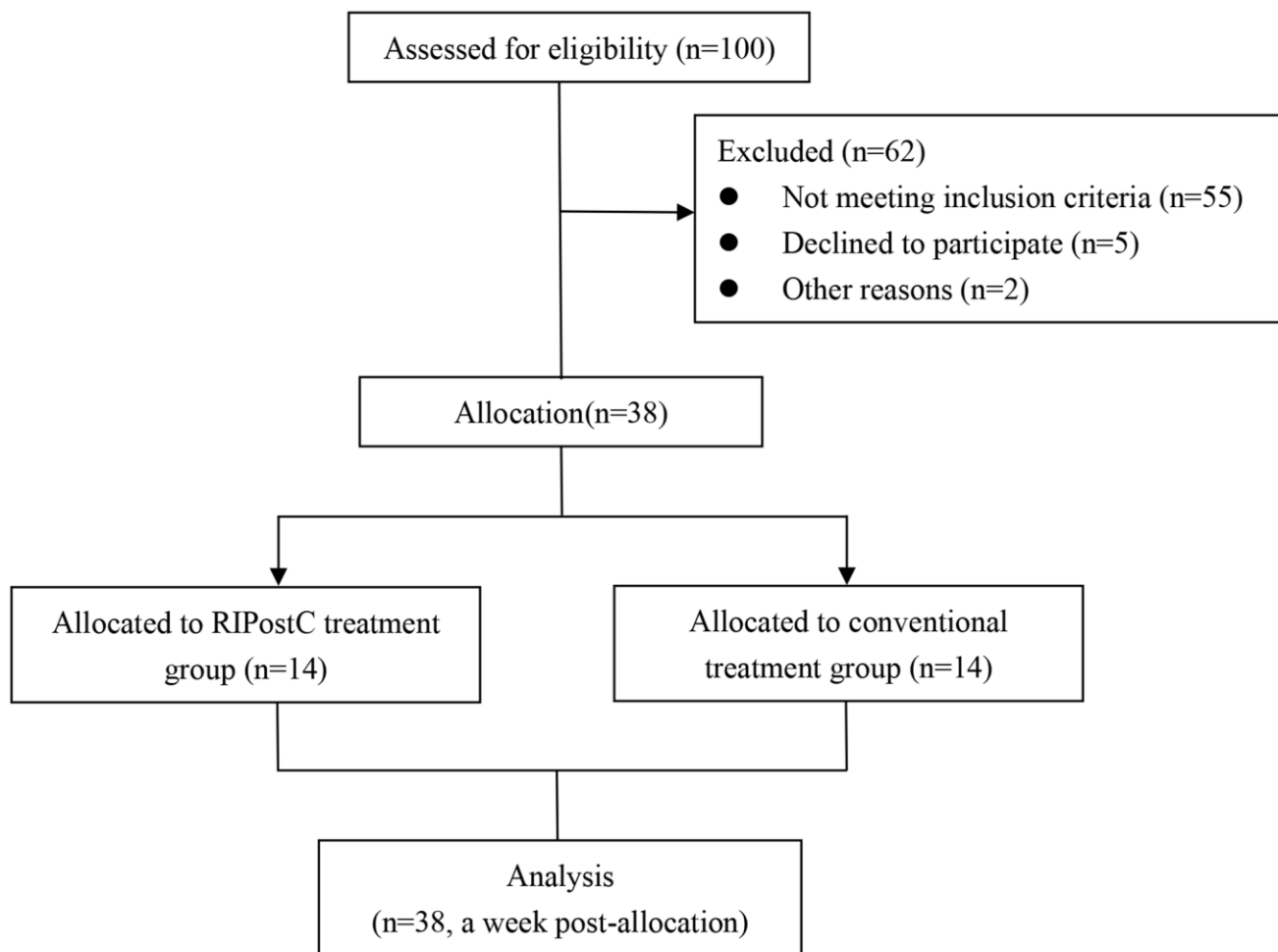

Supplementary Figure 1. CONSORT-style flowchart of the study.

Supplement: Supplementary Figure 1 [file aging-15-204751-s001.pdf]
